# Perturbed Beta-Cell Function and Lipid Profile After Early Prenatal Dexamethasone Exposure in Individuals Without CAH

**DOI:** 10.1210/clinem/dgaa280

**Published:** 2020-04-20

**Authors:** Lena Wallensteen, Leif Karlsson, Valeria Messina, Anna Nordenström, Svetlana Lajic

**Affiliations:** Department of Women’s and Children’s Health, Karolinska Institutet, Pediatric Endocrinology Unit, Karolinska University Hospital, Stockholm, Sweden

**Keywords:** prenatal treatment, dexamethasone, congenital adrenal hyperplasia, metabolism, glucose homeostasis

## Abstract

**Background:**

Prenatal treatment with dexamethasone (DEX) reduces virilization in girls with congenital adrenal hyperplasia (CAH). The treatment is effective but may result in long-lasting adverse effects. In this study we explore the effects of DEX on metabolism in individuals not having CAH but treated with DEX during the first trimester of fetal life.

**Method:**

All DEX-treated participants (n = 40, age range 5.1-26.4 years) and controls (n = 75, age range 4.5-26.6 years) were assessed with fasting blood samples to measure blood count, renal function, glucose homeostasis, and serum lipid profiles.

**Results:**

There were no significant differences between DEX and control participants for birth parameters, weight and height, or body mass index at the time of testing. Analyzing the entire cohort, we found no significant effects of DEX on blood count, renal function, or serum lipid profiles. However, a lower HOMA-β index in the DEX-treated individuals (*U = *893.0; *P* = 0.049) was observed. Post hoc analyses revealed an effect in girls (*U = *152.5; *P* = 0.024) but not in boys (*U = *299.5; *P* = 0.550). The effect on HOMA-β persisted (*U = *117.5; *P* = 0.048) after analyzing data separately in the participants < 16 years of age. In addition, we observed higher plasma glucose levels (*F =* 14.6; *P* = 0.001) in the DEX-treated group. The participants ≥ 16 years of age in the DEX-treated group had significantly higher total plasma cholesterol (*F =* 9.8; *P* = 0.003) and higher low-density lipoprotein cholesterol levels (*F =* 7.4; *P* = 0,009).

**Conclusion:**

Prenatal DEX exposure in early pregnancy has negative effects on beta-cell function and lipid profile in individuals without CAH already at a young age.

Since the 1980s, prenatal dexamethasone (DEX) treatment has been offered to pregnant women at risk of having a child with classic congenital adrenal hyperplasia (CAH) to reduce virilization in an affected female fetus (1-4). The treatment has to be initiated at about gestational weeks 6 to 8 to be effective, implicating that the treatment has to start before the genotyping of the fetus is possible. Consequently, most of the treated children (7 of 8) will be treated unnecessarily during the first trimester of fetal life. Although early fetal sex-typing (SRY detection) using cell-free fetal DNA from maternal blood is an option today to avoid prenatal treatment of boys, it does not eliminate the unnecessary treatment in healthy girls (4). Controversies about the safety of this treatment have prompted the Endocrine Society to state that prenatal DEX treatment should only be offered within a clinical study with written informed consent and that includes transparent and clear information about the potential risks (5). PREDEX, the Swedish long-term follow-up study was established in 1999 to investigate the possible extended effects of prenatal glucocorticoid (GC) treatment in the context of CAH.

We have previously shown the negative effects of prenatal DEX treatment on cognition, especially working memory (6, 7). In an extended study we also identified a sex-dimorphic effect in healthy, first trimester-treated children, with a more pronounced negative effect observed in girls (8). Moreover, our group has recently identified differences in DNA methylation associated with first-trimester DEX treatment in peripheral CD4+ T-cells, which also seems to affect females to a higher extent. These findings make early fetal sex-typing, which aims to treat only female fetuses, less of an advantage (9).

To our knowledge, there is only 1 recent study on metabolic effects after first-trimester DEX treatment in the context of CAH but it only evaluated glucose metabolism (10). Riveline and colleagues found an impaired beta-cell function and subsequent lower insulin secretion at adult age in DEX-treated individuals (10). Long-term follow-up studies are thus necessary to investigate the possibility of negative effects on several aspects of metabolism that are the consequence of early prenatal DEX exposure.

The effects of prenatal GCs on postnatal glucose homeostasis have been studied in different animal species, including primates, although primarily assessing late gestation exposure (11, 12). Higher prenatal doses of GCs, given in mid-gestation and onward, have been associated with fewer and smaller insulin-producing β-cells in the pancreas of the vervet monkey offspring at 8 months of age. Fasting insulin and glucose levels were also higher, as well as increased blood glucose levels after an oral glucose tolerance test (OGTT) (11). Similar outcomes have been seen in humans. In a 30-year follow-up study in individuals born preterm and treated with betamethasone (BETA) during late gestation, the BETA-exposed group had higher insulin levels at 30 minutes (min) and lower glucose levels at 120 min than controls after an OGTT (13). Studies assessing the child’s response to prenatal GC treatment for maternal allergies, asthma, or autoimmune disorders have identified an increase in endocrine and metabolic disorders (13, 14). One study demonstrated a small but significant increase in the incidence of both type 1 and type 2 diabetes (hazard ratio [HR] 1.2 and 1.5, respectively) (14).

Studies investigating the impact of endogenous GC exposure (e.g., maternal stress) in children found that at 15 years of age the children had higher insulin levels and that the levels were also related to the level of stress exposure, as rated by the mother during her pregnancy (15). Contrary to these results, a Swedish study examining young adults (age 14-26 years) treated prenatally with BETA during late gestation reported no effect on body mass index (BMI), blood pressure, blood lipids, cortisol levels, or insulin resistance (16).

In this study, we investigate the long-term metabolic effects of about 6 weeks of DEX treatment during the latter part of the first trimester of fetal life, in children at risk of CAH but later found not to be affected with CAH. We present data on peripheral blood cell counts, glucose homeostasis, lipid profiles, and renal function in DEX-treated individuals compared with age- and sex-matched population controls.

## Methods

### Study cohort

In this retrospective, case-control study we have included all mothers that were DEX-treated during early pregnancy due to the risk of having a child with classic CAH. During 1984-2010, 77 at-risk pregnancies were treated with DEX in Sweden. Four of the pregnancies resulted in miscarriage or termination. In total, 57 (78%) of the 73 DEX-treated children did not have CAH and were potential participants. Six (8%) of the 73 treated mothers (7 pregnancies) did not respond to our follow-up request within the PREDEX study (8). In addition, 7 families declined participation (7 pregnancies) and 1 child had died in an accident at a young age. Of the 42 (58%) remaining (non-CAH) children eligible for inclusion, 2 declined blood sampling but participated in other tests. Thus, 40 of the DEX-treated children and young adults (age range, 4-26 years) were included in the follow-up of the cases treated during first trimester (participation rate 71%).

Controls matched for sex and age were identified through the Swedish civil registration and study information, and invitation letters were sent to 394 individuals in the Stockholm County area. In all, 100 controls accepted to participate in the study, 62 actively declined participation, and the rest did not respond to the invitation. Of the positive responders, 25 declined blood sampling but participated in other tests of the PREDEX study. The participation rate for the control group was thus 19%. The reasons for refusal/nonresponding among controls are not known, but we speculate that the extensive study protocol of the PREDEX study might be one reason. All minors were contacted through their parents by an invitational letter and a subsequent phone call; all the adult participants were contacted directly. Young adults prenatally treated with DEX were contacted only after initial approval from their mothers. All participants, and their mothers, gave their written informed consent to be part of the study as well as consent for us to retrieve clinical data from case files. If the child was under the age of 18 years, the parents signed the consent for the child. Each participant was compensated for participating in the study (50 Euro plus travel expenses). The study was approved by the Regional Ethics Committee in Stockholm (Dnr 99–153).

In summary, 115 individuals were included in the study: 40 DEX-treated individuals (18 females, 22 males) and 75 population controls (35 females, 40 males). The average test age was 16.3 ± 6.2 years. The DEX and control groups did not differ in gestational age, birth length, or birth weight, nor did they differ in age, height, weight or BMI at the time of the blood sampling (all *P* > 0.05; Table 1). All mothers were treated with DEX during the second half of the first trimester, starting at approximately gestational week 6 (6.5 ± 1.1 weeks). The mothers were given a dose of 20 µg/kg/d and treatment was terminated after the genotyping results were obtained from the chorionic villous sampling, which was about gestational week 13 (13.0 ± 2.2 weeks). The mean duration of the treatment was 6.3 ± 2.2 weeks, with no significant differences in treatment length between the DEX-treated boys and girls. Maternal BMI before the pregnancy (*F =* 0.05; *P* = 0.826) and maternal age at parturition did not differ between the study groups (*F =* 0.81; *P* = 0.372) (Table 1).

**Table 1.** Demographic Data for the DEX-Treated Individuals and Controls and Maternal Data

	DEX (female)	C (female)	DEX (male)	C (male)	*P* (DEX)	*P* (DEX × sex)
N	18	35	22	40		
Age, years	16.0 (6.5)	18.2 (4.8)	14.4 (6.9)	15.8 (6.6)	*F =* 2.2 *P =* 0.137	*F =* 0.1 *P =* 0.762
Height, cm	152.5 (21.4)	163.6 (13.3)	156.2 (29.0)	157.4 (26.5)	*F =* 1.8 *P =* 0.187	*F =* 1.2 *P =* 0.283
Weight, kg	49.6 (20.8)	58.8 (16.5)	51.8 (27.4)	53.3 (25.6)	*F =* 1.4 *P =* 0.242	*F =* 0.7 *P =* 0.402
BMI	20.2 (5.5)	21.5 (4.2)	19.4 (4.3)	20.0 (4.5)	*F =* 1.0 *P =* 0.311	*F =* 0.1 *P =* 0.709
Gestational age, weeks	40.0 (1.5)	38.6 (3.2)	39.5 (1.3)	39.8 (2.1)	*F =* 1.6 *P =* 0.205	*F =* 3.3 *P =* 0.073
Birth weight, g	3392.8 (532.5)	3272.6 (615.0)	3627.9 (515.2)	3559.4 (613.5)	*F =* 0.7 *P =* 0.414	*F =* 0.1 *P =* 0.823
Birth weight, z-score	-1,11 (1,51)	-0,55 (1,29)	-0,77 (1,44)	-0,65 (1,37)	*F =* 1.5 *P =* 0.224	*F =* 0.7 *P =* 0.419
Birth length, cm	49.8 (2.7)	49.0 (3.2)	50.7 (2.2)	50.7 (2.4)	*F =* 0.8 *P =* 0.384	*F =* 0.5 *P =* 0.464
Birth length, z-score	-0,33 (1,38)	-0,18 (1,24)	-0,11 (1,28)	-1,10 (5,55)	*F =* 0.4 *P =* 0.528	*F =* 0.7 *P =* 0.399
Maternal age, at parturition, years	31.78 (5,05)	31.03 (4.83)	32.61 (4.97)	31.61 (4.90)	*F =* 0.805 *P =* 0.372	*F =* 0.016 *P =* 0.899
Maternal BMI, before pregnancy	23.12 (4.16)	23.51 (3.67)	24.11 (4.62)	24.15 (3.96)	*F =* 0.049 *P =* 0.826	*F =* 0.034 *P =* 0.854

Data are presented as mean ± 1 SD for age, height, weight and BMI at the time of blood sampling; birth parameters are shown for the DEX-treated individuals and controls. Maternal BMI was based on data from 31 DEX-treated mothers and 48 control mothers.

Abbreviations: BMI, body mass index; C, Controls; DEX, DEX-treated individuals.

### Procedure

All participants were instructed to fast from midnight the night before the blood sampling procedure. The following parameters were analyzed: plasma (P)-cystatin C, cystatin C glomerular filtration rate (GFR), urine (U)-albumin/creatinine, P-sodium, P-potassium, blood count, fasting plasma (fP)-glucose, fP-insulin, blood (B)-HbA1c, fasting serum (fS) -triglycerides, fS-total cholesterol, fS-high-density lipoprotein (fS-HDL) and fS-low-density lipoprotein (fS-LDL). The GFR was calculated using the Schwartz bedside (Crea) and CAPA (cystatin C) formulas (17-19). Beta-cell function and insulin resistance were assessed using the homeostatic model assessment (HOMA-β and HOMA-IR, respectively) (20, 21). All blood samples were analyzed at an accredited laboratory (Department of Clinical Chemistry, Karolinska University Hospital).

### Statistical analyses

All population controls had results within the normal reference range for the Swedish population (22-26) except for 1 young boy who had very low C-peptide and insulin levels. Although his blood glucose and HbA1c levels were within the normal range, we decided to exclude this individual from the analyses of glucose homeostasis.

All variables were tested for normality using the Shapiro-Wilks test while homogeneity of variance was tested with Levene’s test. If assumptions of normality were met, a 2-way analysis of variance was performed to test the effect of DEX on the outcome variables with DEX and sex as independent variables and age as the single covariate. Group differences were considered significant at *P* < 0.05. To investigate the effects of metabolic differences before and after puberty we performed all analyses by dividing the cohort into 2 age groups (< 16 years and ≥ 16 years).

The following variables did not meet the assumption of normality and equal variance: leukocytes, insulin, HOMA-β, HOMA-IR, sodium, triglycerides, and LDL/HDL. Accordingly, group differences for these variables were analyzed using the nonparametric Mann-Whitney U test. After splitting the cohort into the 2 age groups, the nonnormally distributed variables in the younger age group were: erythrocytes, C-peptide, insulin, HOMA-β, HOMA-IR, sodium, and triglycerides; in the older group these variables included C-peptide, insulin, HOMA-β, HOMA-IR, triglycerides, and LDL/HDL.

All statistical analyses were performed using SPSS IBM 24.0 software (SPSS, Armonk, NY).

## Results

### Blood count

Analyzing the data for all DEX-treated individuals and their controls we did not observe any significant effects of DEX treatment on blood count (all *P* > 0.05), irrespective of age (Table 2). However, when the study group was divided into age groups (< 16 and ≥ 16 years), a significant effect of DEX was observed for leukocyte levels in the younger cohort with higher levels in the treated group (*U = *301.5; *P* = 0.003) (Table 3). Table 4 shows there were no effects on blood count in the older age group (all *P* > 0.05).

**Table 2.** Group Averages (mean ± 1 SD) for all Test Scores

	Females		Males		[DEX] ANOVA	[DEX × sex] ANOVA	[DEX] Mann-Whitney U test
	DEX (n = 18)	C (n = 35)	DEX (n = 22)	C (n = 40)	*F* *P*	*F* *P*	*U* *P*
**Blood count**							
Hemoglobin	122.20 (32.57)	130.91 (9.93)	141.36 (13.61)	142.75 (11.60)	*F =* 0.6 *P* = 0.457	*F =* 0.5 *P* = 0.481	
Erythrocytes	4.638 (0.408)	4.547 (0.293)	4.914 (0.327)	4.990 (0.291)	*F =* 0.2 *P* = 0.649	*F =* 2.1 *P* = 0.154	
Leukocytes^a^	6.59 (2.32)	5.80 (1.66)	5.90 (1.32)	5.75 (1.22)			*U = 1586*.0 *P* = 0.268
Thrombocytes	253.25 (49.124)	246.24 (44.569)	265.32 (49.952)	257.00 (56.826)	*F =* 0.0 *P* = 0.888	*F =* 0.1 *P* = 0.784	
**Glucose-insulin metabolism**							
Glucose	4.71 (0.29)	4.69 (0.48)	5.04 (0.50)	4.95 (0.48)	*F =* 1.4 *P* = 0.247	*F =* 0.2 *P* = 0.683	
HbA1c	32.47 (2.53)	31.24 (2.62)	31.60 (2.62)	31.31 (2.04)	*F =* 1.5 *P* = 0.228	*F =* 1.0 *P* = 0.326	
C-peptide	0.56 (0.19)	0.69 (0.24)	0.58 (0.25)	0.58 (0.22)	*F =* 0.6 *P* = 0.433	*F =* 2.3 *P* = 0.134	
Insulin^a^	7.95 (3.45)	10.31 (4.87)	8.03 (5.57)	8.61 (4.49)			U = 1141.0 *P* = 0.086
HOMA-β ^a^	135.25 (72.26)	175.11 (68.71)	112.30 (60.80)	117.30 (53.40)			*U = 893*.0 *P* = 0.049*
HOMA-IR^a^	1.60 (0.71)	2.17 (1.17)	1.98 (1.38)	1.93 (1.16)			*U = 1057*.0 *P* = 0.301
**Renal function**							
Creatinine	54.67 (13.381)	57.30 (13.383)	56.18 (21.952)	61.58 (23.499)	*F =* 0.1 *P* = 0.754	*F =* 1.2 *P* = 0.273	
Sodium^a^	138.9 (1.2)	139.1 (1.8)	139.4 (1.3)	139.3 (3.3)			*U = 1433*.0 *P* = 0.935
Potassium	3.906 (0.22)	3.767 (0.275)	3.895 (0.242)	3.841 (0.330)	*F =* 2.8 *P* = 0.097	*F =* 0.5 *P* = 0.463	
Cystatin C	0.745 (0.09)	0.758 (0.09)	0.796 (0.12)	0.793 (0.08)	*F =* 0.1 *P* = 0.912	*F =* 0.1 *P* = 0.718	
GFR (cystatin-C) (CAPA)	126.1 (20.0)	120.2 (17.4)	119.4 (21.2)	117.3 (16.8)	*F =* 0.2 *P* = 0.691	*F =* 0.1 *P* = 0.811	
GFR (creatinine) (Schwartz bedside)^a^	103.78 (13.45)	108.77 (22.40)	112.60 (28.07)	108.65 (36.10)			*U = 1375*.0 *P* = 0.654
U-albumin	2.56 (0.73)	2.11 (0.72)	2.04 (0.52)	2.02 (0.66)			*U = 873*.0 *P* = 0.885
U-creatinine	14.4 (4.3)	14.2 (7.4)	16.4 (7.8)	16.5 (7.7)	*F =* 1.2 *P* = 0.282	*F =* 0.0 *P* = 0.998	
U-albumin/creatinine^a^	0.81 (0.45)	0.83 (0.80)	0.52 (0.41)	0.77 (1.31)			*U = 786*.5 *P* = 0.874
**Blood lipids**							
Triglycerides^a^	0.76 (0.47)	0.65 (0.30)	0.67 (0.34)	0.70 (0.30)			*U = 1387*.5 *P* = 0.654
Cholesterol	4.57 (0.75)	4.24 (0.70)	4.13 (0.82)	4.00 (0.64)	*F =* 3.0 *P* = 0.085	*F =* 0.6 *P* = 0.437	
HDL	1.66 (0.29)	1.53 (0.31)	1.40 (0.26)	1.37 (0.31)	*F =* 1.4 *P* = 0.246	*F =* 0.6 *P* = 0.428	
LDL	2.95 (0.72)	2.40 (0.60)	2.41 (0.81)	2.33 (0.61)	*F =* 1.4 *P* = 0.243	*F =* 0.2 *P* = 0.655	
LDL/HDL^a^	1.66 (0.58)	1.64 (0.62)	1.84 (0.76)	1.81 (0.65)			*U = 1434*.0 *P* = 0.956

F statistics and *P* values for the main effect of DEX and the interaction between DEX and sex are presented for the variables that were normally distributed. For nonnormally distributed variables, *P* values and U statistics (Mann-Whitney U test) are presented. DEX-treated individuals had significantly lower HOMA-β levels than untreated controls.

Abbreviations: ANOVA, analysis of variance; BMI, body mass index; C, Controls; CAPA, Caucasian, Asian, pediatric and adult; DEX, DEX-treated individuals; GFR, glomerular filtration rate; HDL, high-density lipids; HOMA-β, homeostatic model assessment for beta-cell function; HOMA-IR, homeostatic model assessment for insulin resistance; LDL, low-density lipids.

^a^Nonparametric statistics were used.

**Table 3.** Group Averages (mean ± 1 SD) for all Test Scores in the Age Group < 16 years

	Females		Males		[DEX] ANOVA	[DEX × sex] ANOVA	[DEX] Mann- Whitney U test
	DEX (n = 7)	Controls (n = 8)	DEX (n = 12)	Controls (n = 17)	*F* *P*	*F* *P*	*U* *P*
**Blood count**							
Hemoglobin	127.7 (6.5)	129.3 (8.2)	132.0 (9.0)	134.1 (11.1)	*F =* 0.0 *P* = 0.893	*F =* 0.5 *P* = 0.491	
Erythrocytes^a^	4.78 (0.44)	4.56 (0.30)	4.72 (0.22)	4.93 (0.36)			*U = 182*.0 *P* = 0.385
Leukocytes	6.65 (2.89)	5.24 (2.08)	6.12 (1.06)	5.25 (0.93)	*F =* 0.9 *P* = 0.771	*F =* 4.5 *P* = 0**.040**	
Thrombocytes	268.2 (52.0)	271.0 (53.8)	295.9 (38.9)	275.9 (60.9)	*F =* 0.01 *P* = 0.916	*F =* 1.3 *P* = 0.258	
**Glucose-insulin metabolism**							
Glucose	4.77 (0.21)	4.44 (0.58)	4.95 (0.36)	4.71 (0.28)	*F =* 14.6 *P* = 0**.001**	*F =* 2.0 *P* = 0.162	
HbA1c	33.00 (2.10)	31.71 (1.98)	32.00 (2.70)	31.54 (2.18)	*F =* 1.1 *P* = 0.301	*F =* 0.2 *P* = 0.621	
C-peptide^a^	0.41 (0.1)	0.50 (0.24)	0.50 (0.21)	0.48 (0.19)			*U = 196*.5 *p* = 0.578
Insulin^a^	5.77 (1.56)	7.60 (4.74)	7.18 (5.13)	7.04 (3.90)			*U = 198*.5 *P* = 0.613
HOMA-β ^a^	86.65 (20.57)	148.91 (44.34)	101.53 (57.55)	114.68 (51.23)			*U = 117*.5***P* = 0.048**
HOMA-IR^a^	1.16 (0.33)	1.59 (1.17)	1.71 (1.24)	1.5 (0.90)			*U = 194*.0 *P* = 0.978
**Renal function**							
Creatinine	41.4 (5.8)	39.6 (7.9)	37.7 (8.2)	37.8 (9.1)	*F =* 1.6 *P* = 0.215	*F =* 1.2 *P* = 0.284	
Sodium^a^	139.0 (1.0)	138.5 (1.5)	138.8 (1.1)	138.0 (2.8)			*U = 267*.5 *P* = 0.464
Potassium	3.91 (0.12)	3.79 (0.18)	3.92 (0.22)	3.81 (0.21)	***F =* 5.5** ***P* = 0.025**	*F =* 0.1 *P* = 0.765	
Cystatin C	0.717 (0.095)	0.726 (0.089)	0.798 (0.116)	0.771 (0.067)	*F =* 0.1 *P* = 0.773	*F =* 0.3 *P* = 0.579	
GFR (cystatin C; CAPA)	139.9 (19.6)	133.8 (18.0)	125.3 (21.4)	127.3 (14.2)	*F =* 0.0 *P* = 0.988	*F =* 0.3 *P* = 0.613	
GFR (creatinine; Schwartz bedside)	114.66 (8.83)	135.81 (18.76)	133.26 (17.55)	134.39 (35.44)	*F =* 2.2 *P* = 0.144	*F =* 2.8 *P* = 0.103	
U-albumin^a^	12.0 (3.8)	12.2 (4.4)	12.5 (4.6)	10.8 (4.0)			*U = 179*.5 *P* = 0.068
U-creatinine^a^	12.0 (3.8)	12.2 (4.4)	12.5 (4.6)	10.8 (3.9)			*U = 235*.5 *P* = 0.488
U-Alb/Crea ratio^a^	1.24 (0.17)	0.72 (0.32)	0.72 (0.47)	1.33 (2.00)			*U = 155*.0 *P* = 0.372
**Blood lipids**							
Triglycerides^a^	0.60 (0.49)	0.50 (0.16)	0.51 (0.24)	0.61 (0.26)			*U = 174*.0 *P* = 0.209
Cholesterol	4.3 (0.85)	4.18 (0.47)	3.84 (0.71)	4.30 (0.65)	*F =* 0.3 *P* = 0.563	*F =* 2.4 *P* = 0.134	
HDL	1.64 (0.39)	1.58 (0.15)	1.50 (0.25)	1.45 (0.19)	*F =* 0.6 *P* = 0.438	*F =* 0.0 *P* = 0.853	
LDL	2.43 (0.65)	2.38 (0.47)	2.07 (0.57)	2.57 (0.63)	*F =* 1.0 *P* = 0.321	*F =* 2.3 *P* = 0.136	
LDL/HDL-ratio	1.59 (0.62)	1.51 (0.35)	1.46 (0.30)	1.81 0.52)	*F =* 0.6 *P* = 0.448	*F =* 2.2 *P* = 0.150	

F statistics and *P* values for the main effect of DEX and the interaction between DEX and sex are presented for normally distributed variables. For nonnormally distributed variables, *P* values and U statistics (Mann-Whitney U test) are presented. DEX-treated individuals had significantly lower HOMA-β and higher plasma glucose and plasma potassium levels than untreated controls. They also had a higher blood leukocyte count.

Abbreviations: ANOVA, analysis of variance; BMI, body mass index; C, Controls; CAPA, Caucasian, Asian, pediatric and adult; DEX, DEX-treated individuals; GFR, glomerular filtration rate; HDL, high-density lipids; HOMA-β, homeostatic model assessment for beta-cell function; HOMA-IR, homeostatic model assessment for insulin resistance; LDL, low-density lipids.

^a^Nonparametric statistics were used.

**Table 4.** Group Averages (mean ± 1 SD) for all Test Scores in the Age Group ≥ 16 years

	Females		Males		[DEX] ANOVA	[DEX × sex] ANOVA	[DEX] Mann-Whitney U test
	DEX (n = 11)	C (n = 27)	DEX (n = 10)	C (n = 23)	*F* *P*	*F* *P*	*U* *P*
**Blood count**							
Hemoglobin	132.7 (9.9)	131.3 (10.4)	152.6 (8.7)	149.1 (7.0)	*F =* 1.0 *P* = 0.332	*F =* 0.2 *P* = 0.690	
Erythrocytes	4.55 (0.38)	4.54 (0.30)	5.15 (0.28)	5.03 (0.23)	*F =* 0.6 *P* = 0.444	*F =* 0.5 *P* = 0.491	
Eeukocytes	6.56 (2.08)	5.95 (1.55)	5.64 (1.59)	6.11 (1.30)	*F =* 0.3 *P* = 0.868	*F =* 1.7 *P* = 0.198	
Thrombocytes	244.3 (47.8)	240.0 (40.6)	228.6 (35.1)	243.0 (50.5)	*F =* 0.2 *P* = 0.657	*F =* 0.7 *P* = 0.409	
**Glucose-insulin metabolism**							
Glucose	4.67 (0.33)	4.77 (0.40)	5.16 (0.65)	5.11 (0.52)	*F =* 0.0 *P* = 0.835	*F =* 0.3 *P* = 0.603	
HbA1c	32.2 (2.8)	31.1 (2.8)	31.0 (2.1)	31.2 (2.0)	*F =* 0.6 *P* = 0.434	*F =* 0.7 *P* = 0.402	
C-peptide^a^	0.66 (0.16)	0.75 (0.20)	0.68 (0.26)	0.64 (0.23)			*U = 463*.5 *P* = 0.513
Insulin^a^	9.34 (3.65)	11.15 (4.69)	9.05 (6.16)	9.68 (4.63)			*U = 416*.0 *P* = 0.251
HOMA-β ^a^	164.4 (77.1)	182.4 (73.2)	127.1 (65.9)	119.3 (56.2)			*U = 372*.0*P* = 0.616
HOMA-IR^a^	1.86 (0.76)	2.36 (1.13)	2.36 (1.55)	2.23 (1.25)			*U = 375*.0 *P* = 0.560
**Renal function**							
Creatine	63.1 (9.1)	63.0 (9.1)	78.4 (6.0)	79.1 (12.7)	*F =* 0.0 *P* = 0.854	*F =* 0.0 *P* = 0.828	
Sodium	138.8 (1.3)	139.3 (1.8)	140.2 (1.1)	140.5 (3.3)	*F =* 0.3 *P* = 0.578	*F =* 0.0 *P* = 0.861	
Potassium	3.90 (0.27)	3.76 (0.30)	3.86 (0.27)	3.87 (0.41)	*F =* 0.7 *P* = 0.401	*F =* 0.8 *P* = 0.384	
Cystatin C	0.77 (0.08)	0.77 (0.09)	0.79 (0.12)	0.81 (0.09)	*F =* 0.2 *P* = 0.658	*F =* 0.1 *P* = 0.807	
GFR (cystatin C; CAPA)	116.4 (14.1)	116.2 (15.3)	112.4 (19.8)	108.5 (13.7)	*F =* 0.3 *P* = 0.609	*F =* 0.2 *P* = 0.669	
GFR (creatinine; (Schwartz bedside)	95.85 (11.18)	99.76 (15.16)	85.04 (7.15)	84.35 (11.61)	*F =* 0.1 *P* = 0.916	*F =* 0.2 *P* = 0.623	
U-albumin	10.50 (8.50)	11.81 (10.37)	7.14 (3.62)	8.35 (4.92)			*U = 250*.5 *P* = 0.685
U-creatinine^a^	15.94 (3.99)	14.80 (8.07)	22.26 (8.07)	20.10 (7.29)			*U = 514*.5 *P* = 0.335
U-albumin/creatinine^a^	0.55 (0.35)	0.89 (0.97)	0.29 (0.11)	0.42 (0.23)			*U = 221*.0 *P* = 0.380
**Blood lipids**							
Triglycerides^a^	0.87 (0.44)	0.70 (0.32)	0.83 (0.37)	0.76 (0.32)			*U = 604*.5 *P* = 0.316
Cholesterol	4.75 (0.67)	4.26 (0.77)	4.45 (0.85)	3.78 (0.55)	*F =* 9.8 ***P* = 0.003**	*F =* 0.2 *P* = 0.644	
HDL	1.67 (0.22)	1.52 (0.35)	1.28 (0.22)	1.30 (0.36)	*F =* 0.8 *P* = 0.361	*F =* 1.0 *P* = 0.330	
LDL	2.69 (0.59)	2.41 (0.63)	2.78 (0.89)	2.16 (0.55)	*F =* 7.4 ***P* = 0.009**	*F =* 1.1 *P* = 0.306	
LDL/HDL^a^	1.71 (0.58)	1.67 (0.68)	2.26 (0.90)	1.82 (0.74)			*U = 591*.5 *P* = 0.233

F statistics and p-values for the main effect of DEX and the interaction between DEX and sex are presented for normally distributed variables. For non-normally distributed variables, p-values and U statistics (Mann-Whitney U test) are presented. DEX-treated individuals showed significantly higher plasma total cholesterol levels and low-density lipid cholesterol.

Abbreviations: ANOVA, analysis of variance; BMI, body mass index; C, Controls; CAPA, Caucasian, Asian, pediatric and adult; DEX, DEX-treated individuals; GFR, glomerular filtration rate; HDL, high-density lipids; HOMA-β, homeostatic model assessment for beta-cell function; HOMA-IR, homeostatic model assessment for insulin resistance; LDL, low-density lipids.

^b^Nonparametric statistics were used.

### Glucose homeostasis

In the whole group, no significant effects of prenatal DEX treatment were observed for levels of glucose, HbA1c, C-peptide, HOMA-IR, and insulin (all *P* > 0.05) (Table 2). However, we did observe a significant effect of DEX on HOMA-β with lower beta-cell function in the treated group (*U = *893.0; *P* = 0.049). When females and males were analyzed separately, the lower HOMA-β values persisted in girls (*U = *152.5; *P* = 0.024) but not in boys (*U = *299.5; *P* = 0.550). When analyzing the 2 age groups separately, a significant effect of DEX was noted in the younger children, with higher glucose in the treated group (*F =* 14.6, *P* = 0.001) (Table 3). The younger children also presented lower HOMA-β values (*U = *117.5; *P* = 0.048) (Table 3), where a post hoc analysis indicated that the effect was driven by a significant effect in the DEX-treated girls (*U = *2.0; *P* = 0.05) but not in the DEX-treated boys (*U = *70; *P* = 0.540).

There were no significant effects of DEX on glucose metabolism in the older age group (all *P* > 0.05) (Table 4).

### Renal function

No significant effects of prenatal DEX treatment were discerned in the whole cohort on measures of renal function (all *P* > 0.05) (Tables 2-4). However, dividing the group based on age revealed a significant effect on potassium concentrations in the younger group (*F =* 5.5; *P* = 0.025), with DEX-treated children exhibiting higher levels of plasma potassium.

### Blood lipids

There were no significant effects of prenatal DEX treatment on blood lipid profiles when the entire DEX-treated cohort was compared with the population controls (all *P* > 0.05) (Table 2).

When the groups of younger and older participants were analyzed separately, a significant effect of DEX was identified in the older age group for cholesterol (*F =* 9.8; *P* = 0.003) and LDL cholesterol (*F =* 7.4; *P* = 0.009) levels (Table 4). In the younger age group, no significant results were detected (all *P* > 0.05) (Table 3).

## Discussion

This study evaluated the effects of early prenatal dexamethasone treatment on the metabolism of children and adults at risk of CAH. We analyzed glucose homeostasis, blood count, renal function, and lipid profiles. None of the DEX-treated participants had CAH, but they had all been exposed to DEX during the first trimester of fetal life for a mean duration of 6 weeks. To investigate whether the metabolic outcome would differ in prepubertal versus postpubertal individuals, analyses were performed in younger (< 16 years) and older (≥ 16 years) participants.

We observed significantly lower HOMA-β levels in the DEX-treated group and evidence for sex-dimorphic effects, with a more pronounced effect on beta-cell function in treated girls. Moreover, this result persisted in children < 16 but not in the youth ≥ 16 years old. HOMA-β is a marker of beta-cell function and is a common test used in clinical practice (27). The current findings indicate that prenatal DEX treatment given during early fetal development affects the activity of the beta cells, which might lead to impaired glucose tolerance. A plausible reason for why we did not observe the same effect in the older age group could be an issue of insufficient power due to the small sample size. An alternative hypothesis is that insulin resistance markedly increases throughout puberty. During puberty, there is a need for more efficient beta-cell function, with any loss of function becoming more evident in this age group (28-30).

The recent study by Riveline et al is congruent with our findings (10). The authors reported that adults who had been treated during the first trimester with DEX had significantly lower insulin secretion compared with controls. They suggested that this could be caused by a decrease in beta-cell mass and function (10) and concluded that the altered response in insulin secretion might cause alterations in glucose tolerance with time. In accord with our finding of lower beta-cell activity, we also observed higher fasting glucose levels in DEX-treated children. The glucose levels were still within the normal range, but the higher levels may become more important as the individuals age (per the above argument).

Altered blood lipid profiles identified as both higher total cholesterol and LDL levels were observed in the DEX-exposed group ≥ 16 years. During childhood and puberty, there is a natural decrease in serum cholesterol and HDL and LDL levels. There are also differences between the sexes in blood lipid profiles, especially in late puberty and adulthood, a difference that is much less evident in prepubertal children (31, 32). Women have higher levels of blood lipids than men. We may hypothesize that the reason for only observing a difference in blood lipids in the older age group is that the mechanism underlying the altered blood lipid homeostasis is an absence of a natural decline in blood lipid levels during puberty. A high cholesterol level, especially LDL, is a well-known risk factor for coronary heart disease (33, 34). If these individuals tend towards impaired glucose tolerance, in addition to higher blood lipids, the risk of coronary heart disease will likely increase (35).

The finding of altered levels of leukocytes in the young cohort remains unexplained, although we could speculate that it has to do with a programming effect of GCs on the immune system. This conjecture is consistent with our previous finding of altered DNA methylation in peripheral T cells (9), in which the effect on DNA methylation was mainly associated with immune functioning and inflammation and may be a reflection of this finding (9).

Potassium levels were higher in the DEX-treated group, which may be attributable to an effect on tubular renal function or due to altered aldosterone synthesis or function. Previously, we (9) observed differential methylation in several genes coding for enzymes involved in GC and mineralocorticoid synthesis (9). Among these was the *CYP11B2* gene that codes for the enzyme aldosterone synthase. The gene harbored differentially methylated CpGs in its promotor region (9).

Data on long-term metabolic effects of prenatal DEX treatment in the context of CAH are limited, with only the data presented here and those recently published by Riveline et al (10). Most human studies assessing prenatal GC effects on children are studying late-gestation exposure, which makes comparison with our results problematic as the different treatment strategies during alternative developmental time windows may affect the fetus differently. Some cautious conclusions may, however, be drawn from studies on experimental animals and human studies in which the fetus is exposed to GC treatment in late gestation or maternal stress. In humans, prenatal exposure to endogenous GC due to maternal stress has been shown to result in impaired glucose tolerance, especially if the exposure is in early- to mid-gestation (36). Moreover, higher stated maternal stress predicted higher insulin levels in the child or young adult (15). Young adults (23-28 years of age) exposed to GC treatment during late gestation because of a risk of being born preterm displayed a significantly lower HOMA-β compared with untreated controls (37), which is in agreement with our results (10). Whether the mechanism is a direct negative effect on the beta-cell mass or whether the effect is also caused by changed insulin sensitivity (13) or an effect of changed homeostasis of the hypothalamic-pituitary-adrenal axis (38) is yet to be determined.

Early prenatal treatment with cortisol or DEX changed the glucose homeostasis in the offspring of adult sheep (39). In another study in which BETA was given during mid-gestation to pregnant ewes, higher insulin levels were noted in adult female offspring after an intravenous glucose tolerance test (40).

## Strengths and Limitations

Our study included a well-characterized cohort of prenatally DEX-treated children, adolescents, and young adults. Although our study included most of the DEX-treated cases in Sweden, the cohort is still relatively small in comparison with what usually is desired in similar observational studies. Thus, it may be that we failed to detect effects (type 2 error) because of a lack of power. In addition, separation by pubertal stage instead of age might have been more optimal for some of the analyses.

## Conclusion

In summary, early prenatal DEX exposure affects the function of the pancreatic beta cell, here identified as lower HOMA-β levels, and alters the lipid profile with higher total cholesterol as well as higher LDL-cholesterol levels in treated subjects. The data has implications for the future use of prenatal treatment in the context of CAH, as the majority of the treated cases that are exposed to DEX antenatally do not benefit from such treatment themselves.

Perturbed beta-cell function and increased LDL-cholesterol levels in combination with existing evidence on the negative cognitive effects observed in individuals treated during the first trimester (8) and the epigenetic differences seen in our cohort (9) increases the concerns of DEX treatment. Can it be justified to expose the majority of the children to benefit the minority, even if the minority would have a large benefit? If prenatal treatment with DEX is considered, the possible long-term consequences on health must be carefully and thoroughly discussed with the parents and treatment should only be performed in the form of a study to ensure careful and thorough follow-up.

## Abbreviations

BETA: betamethasone
BMI: body mass index
CAH: congenital adrenal hyperplasia
CAPA: Caucasian, Asian, pediatric and adult
DEX: dexamethasone
fS: fasting serum
GC: glucocorticoid
GFR: glomerular filtration rate
HbA1c: glycated hemoglobin A1c
HDL: high-density lipoprotein
HOMA-β: homeostatic model assessment for beta-cell function
HOMA-IR: homeostatic model assessment for insulin resistance
LDL: low-density lipoprotein

## Additional Information

***Disclosure Summary*:** Authors have nothing to disclose.

***Data Availability:*** The datasets generated during and/or analyzed during the current study are not publicly available but are available from the corresponding author on reasonable request.
